# On the importance of avoiding shortcuts in applying cognitive models to hierarchical data

**DOI:** 10.3758/s13428-018-1054-3

**Published:** 2018-06-12

**Authors:** Udo Boehm, Maarten Marsman, Dora Matzke, Eric-Jan Wagenmakers

**Affiliations:** 1 Department of Experimental Psychology, University of Groningen, 9712 TS Groningen, The Netherlands; 2 Department of Psychology, University of Amsterdam, 1018 XA Amsterdam, The Netherlands

**Keywords:** Cognitive models, Statistical test, Statistical errors, Bayes factor, Hierarchical Bayesian model

## Abstract

Psychological experiments often yield data that are hierarchically structured. A number of popular shortcut strategies in cognitive modeling do not properly accommodate this structure and can result in biased conclusions. To gauge the severity of these biases, we conducted a simulation study for a two-group experiment. We first considered a modeling strategy that ignores the hierarchical data structure. In line with theoretical results, our simulations showed that Bayesian and frequentist methods that rely on this strategy are biased towards the null hypothesis. Secondly, we considered a modeling strategy that takes a two-step approach by first obtaining participant-level estimates from a hierarchical cognitive model and subsequently using these estimates in a follow-up statistical test. Methods that rely on this strategy are biased towards the alternative hypothesis. Only hierarchical models of the multilevel data lead to correct conclusions. Our results are particularly relevant for the use of hierarchical Bayesian parameter estimates in cognitive modeling.

## Introduction

Quantitative cognitive models are an important tool in understanding the human mind. These models link latent cognitive processes, represented by the models’ parameters, to observable variables, thus allowing researchers to formulate precise hypotheses about the relationship between cognitive processes and observed behavior. To test these hypotheses, researchers fit the model to experimental data from a sample of participants who perform several trials of an experimental task. Although this procedure might seem straightforward, the hierarchical data structure induces a number of subtleties.

For example, the diffusion decision model (DDM; Ratcliff, 1978; Ratcliff et al., 2016) conceptualizes decision-making in terms of seven model parameters that represent different cognitive processes, such as encoding of the response stimulus and response caution. Using these seven model parameters, the DDM describes the response time (RT) distribution that results from repeated performance of a decision-making task. A researcher might, for instance, hypothesize that caffeine leads to faster decision-making due to improved attention. In terms of the DDM, this hypothesis would be described as an increase in the model parameter that represents the speed of stimulus encoding but no change in response caution. To test this hypothesis, the researcher randomly assigns participants either to a group that is given a placebo or to a group that is given caffeine and asks participants to perform several trials of the Eriksen flanker task (e.g., Lorist & Snel, 1997). In the Eriksen flanker task (Eriksen & Eriksen, 1974), participants are presented a central stimulus that is surrounded by two distractors on each side, the flankers. The participants’ task is to respond as quickly as possible to the central stimulus while ignoring the flankers. The researcher subsequently wishes to fit the DDM to participants’ RT data and compare the estimated speed of stimulus processing and response caution between groups (see also White et al., 2011). Complications in modeling these data arise from the fact that the experimental setup leads to a hierarchical data structure, with trials (i.e., repeated measurements) nested within participants. A proper analysis of these data therefore requires a hierarchical implementation of the DDM. However, two common modeling strategies, namely ignoring the hierarchy and taking a two-step analysis approach, do not properly account for the hierarchical data structure.

First, ignoring the hierarchy means that researchers model the data for each participant independently and subsequently pool parameter point estimates across participants for further statistical analyses. A researcher might, for example, fit the DDM independently to each participant’s RT data and enter the resulting parameter estimates into a *t* test or ANOVA-type analysis. In a simpler version of this strategy, researchers compute the mean RT for each participant and subsequently perform statistical inference on the participant means. Although analyses that ignore the hierarchy might be unavoidable if only non-hierarchical implementations of a particular cognitive model are available, such analyses risk statistical biases. As we will show in the present work, ignoring the hierarchy can lead to an underestimation of effect sizes and statistical tests that are biased towards the null hypothesis.

Second, taking a two-step analysis approach means that researchers apply a hierarchical cognitive model to their data and subsequently perform further statistical analyses on the parameter point estimates for individual participants. This strategy is closely linked to the recent development and popularization of hierarchical Bayesian cognitive models (Rouder & Jun, 2005; Rouder et al., 2003; Lindley & Smith, 1972). A hierarchical version of the DDM (Wiecki et al., 2013), for example, assumes that each participant’s RT distribution is characterized by seven DDM parameters; these participant-level parameters are in turn drawn from group-level distributions that are characterized by a set of parameters of their own. Finally, in an ideal application, the effect of the experimental manipulation is described by the difference between group-level parameters, most commonly expressed as a standardized effect size. One favorable property of such a hierarchical model is that parameter estimates for individual participants are informed by the parameter estimates for the rest of the group; less reliable estimates are more strongly pulled towards the group mean, a property that is referred to as shrinkage (Gelman et al., 2013; Efron & Morris, 1977). Shrinkage reduces the influence of outliers on group-level estimates and at the same time improves the estimation of individual participants’ parameters. In clinical populations, for instance, individual estimates are often associated with considerable variability, as only few participants can be recruited and little time is available for testing so that hierarchical methods need to be employed to obtain reliable estimates of group-level parameters (Krypotos et al., 2015).

Due to the shrinkage property, hierarchical Bayesian methods provide estimates of individual participants’ parameters with the smallest estimation error (Efron & Morris, 1977), and it therefore seems prudent also to base inferences about groups on hierarchical Bayesian parameter estimates for individuals. This might seem to suggest a two-step approach where parameter point estimates obtained from a hierarchical Bayesian model are used in a follow-up frequentist test. Researchers might furthermore feel compelled to use a two-step approach because they are more familiar with frequentist methods, because the journal requires authors to report *p* values, or because the software for fitting a hierarchical Bayesian version of a particular cognitive model is not sufficiently flexible to carry out the desired analysis. However, tempting as a two-step approach might seem, it is fraught with difficulties. Although hierarchical Bayesian methods provide the best estimates for individuals’ parameters on average (Farrell & Ludwig, 2008; Rouder et al., 2003), if used in statistical tests such hierarchical estimates can potentially lead to inflated effect sizes and test statistics (see e.g., Mislevy, 1991; Mislevy et al., 1992 for a more complete discussion of problems associated with a two-step analysis approach).

### Relevance

Hierarchically structured data are ubiquitous in cognitive science and analysis strategies that either ignore the hierarchy or take a two-step approach are highly prevalent in practice. For example, of the most recent 100 empirical papers in *Psychonomic Bulletin & Review’s* Brief Report section (volume 23, issues 2-4), 93 used a hierarchical experimental design. Of these 93 papers, 74 used a statistical analysis that was based on participant means and thus ignored the hierarchical data structure. That means that the statistical results in about 80*%* of these 93 papers might be biased due to an incorrect analysis strategy. Ignoring the hierarchy is also common in more sophisticated analyses that are based on cognitive models (e.g., Beitz et al.,, 2014; Cooper et al.,, 2015; Epstein et al., 2006; Kieffaber et al., 2006; Kwak et al.,, 2014; Leth-Steensen et al.,, 2000; Penner-Wilger et al.,, 2002; Ratcliff et al.,, in press; Ratcliff et al., 2004; Ratcliff et al.,, 2001). The frequency with which researchers take a two-step approach is harder to assess because the number of studies that use hierarchical Bayesian cognitive models is still relatively low. Nevertheless, a number of authors from different areas of psychology have recently taken a two-step approach to analyzing their data (Ahn et al., 2014; Badre et al., 2014; Chan et al., 2013; Chevalier et al., 2014; Matzke et al., 2015; Vassileva et al., 2013; Driel et al., 2014; Zhang et al., 2016; Zhang & James, 2014), which suggests that this analysis approach and the associated statistical biases might become more prevalent in the literature as hierarchical Bayesian models gain popularity.

As pointed out above, there are compelling pragmatic reasons why researchers might ignore the hierarchy or take a two-step analysis approach. In addition, cognitive models are often difficult to estimate per se (e.g., Turner et al., 2013) and introducing a hierarchical structure into the model might yield an overly complex model that cannot be estimated reliably in practice. However, researchers should to be aware of and acknowledge the potential biases associated with these strategies. Although the biases associated with each strategy tend to become negligible if sufficient data is available, exactly how much data are needed to render statistical biases inconsequential will depend on the specific cognitive model. It is therefore important to understand the general mechanisms and potential magnitude of statistical biases introduced by these analysis approaches.

The goal of the present work is to illustrate how statistical results can be biased by analyses of hierarchical data that (1) ignore the hierarchy, or (2) take a two-step approach. To this end, we will discuss five prototypical analysis strategies, two of which correctly represent the data structure, and three which commit one or the other mistake. We will base our discussion of the different analysis strategies on a model that assumes normal distributions on the group-level and on the participant-level. Although this model is far removed from the complexity typically found in cognitive models, its structure simplifies the theoretical treatment of the different modeling strategies. These results can then be easily generalized to more complex, cognitive models.

We begin with a brief discussion of some well-established theoretical results that explain how the different analysis strategies will impact statistical inference. We then illustrate the practical consequences of these theoretical results in a simulation study. Nevertheless, to anticipate our main conclusions, ignoring the hierarchy generally biases statistical tests towards the null hypothesis. Taking a two-step analysis approach, on the other hand, biases tests towards the alternative hypothesis. In addition, Bayesian hypothesis tests that ignore the hierarchy show an overconfidence bias; when tests favor the alternative hypothesis, they indicate stronger evidence for the alternative hypothesis than warranted by the data, and when tests favor the null hypothesis, they indicate stronger evidence for the null hypothesis than warranted by the data.

## Part I: Statistical background

In this section, we will provide a basic technical account of the different analysis strategies and how they impact statistical inference (see Box & Tiao, 1992, for a similar discussion). Readers who are not interested in these details can skip ahead to the section “Consequences for five different analysis strategies”. For the sake of simplicity, we will assume that all data are normally distributed. Nevertheless, the basic mechanisms discussed here also hold for more complex models.

In a typical experimental setup, for each participant *i*, *i* = 1,…,*N*, a researcher obtains a number of repeated measurements *j*, *j* = 1,…,*K*, of a variable of interest, such as pupil dilation, test scores, or skin conductance. These trial-level measurements are prone to participant-level variance, that is, given the participant’s true mean *𝜃*_*i*_, the observations *x*_*ij*_ are independent and normally distributed:

1 $$ x_{ij} \sim \mathcal{N}\left( \theta_{i},\sigma^{2}\right), $$

where *σ*^2^ is the participant-level variance.^1^ Moreover, given the group-level mean *μ*, the true participant-level means *𝜃*_*i*_ for different participants are independent and normally distributed:

2 $$ \theta_{i} \sim \mathcal{N}\left( \mu,\tau^{2}\right) $$

with variance *τ*^2^. When *τ*^2^ is large, this indicates that participants are relatively heterogeneous (Shiffrin et al., 2008).

Researchers are usually interested in making statements about the group-level mean *μ* for different experimental groups. However, the group-level mean is not directly observable and therefore needs to be estimated. The simplest estimate for the group-level mean would be the average of participants’ true means, $\bar {\theta }$. Because participants’ true means vary around the group-level mean with variance *τ*^2^, the average $\bar {\theta }$ has some uncertainty associated with it. Moreover, the true participant means *𝜃*_*i*_ themselves are also unobservable, and therefore need to be estimated. A simple point estimate for each participant’s true mean is the average of the person’s repeated measurements, $\bar {x}_{i}$. Because the repeated measurements vary around the person’s true mean, the average $\bar {x}_{i}$ has sampling variance *σ*^2^/*K* associated with it. Consequently, there are two sources of variance that influence the distribution of the $\bar {x}_{i}$ around the group-level mean *μ*, namely the group-level variance *τ*^2^ and the sampling variance *σ*^2^/*K*:

3 $$ \bar{x}_{i} \sim \mathcal{N}\left( \mu, \tau^{2} + \frac{\sigma^{2}}{K}\right). $$

Ignoring either of these variance components can considerably bias researchers’ analyses, as we will discuss in the next sections. We will first turn to the problem of ignoring the hierarchical data structure, which leads to an overestimation of the group-level variance, before we discuss the problem of a two-step analysis approach, which leads to an underestimation of the group-level variance.

### First faulty method: Ignoring the hierarchy

The first faulty analysis method that is highly prevalent in current statistical practice is ignoring the hierarchical data structure, which is equivalent to missing a random effect on the participant-level. The underlying mechanism is common to both Bayesian and frequentist analyses and leads to an overestimation of the group-level variance. When researchers ignore the hierarchy, they base their analysis on participants’ sample averages $\bar {x}_{i}$ and equate these with participants’ true means *𝜃*_*i*_. This tacitly implies that the variance of the $\bar {x}_{i}$ is assumed to equal the group-level variance *τ*^2^. However, the variance of the $\bar {x}_{i}$ is in fact the sum of the true group-level variance *τ*^2^ and the sampling variance *σ*^2^/*K* (see Eq. 3), and as a result researchers overestimate the group-level variance by *σ*^2^/*K*. Although the problem is negligible when the number of trials per participant *K* is large, the sampling variance *σ*^2^/*K* is usually unknown and it is unclear for what size of *K* the influence of the sampling variance becomes negligible relative to the group-level variance. Moreover, the rate at which the overestimation of the group-level variance decreases with increasing *K* will also depend on the specific cognitive model and will be considerably larger for some models than for others.

### Second faulty method: Two-step analyses

The second faulty analysis method regularly seen in the recent literature is taking a two-step approach. Much as ignoring the hierarchy, this method is detrimental to the validity of statistical conclusions but has the opposite effect. While ignoring the hierarchy leads to an overestimation of the group-level variance, taking a two-step approach leads to an underestimation of the group-level variance. Here we focus on the analysis strategy where researchers obtain point estimates from a hierarchical Bayesian model and use participant-level estimates in a non-hierarchical follow-up test. However, the same problems can be expected to befall analyses that use participant-level point estimates from a hierarchical frequentist model in a non-hierarchical follow-up test.

A two-step analysis is based on an appropriately specified hierarchical Bayesian model. Given the experimental setup outlined above, the appropriate hierarchical model postulates that repeated measurements for each participant are normally distributed around a true mean ($x_{ij} \sim \mathcal {N}\left (\theta _{i},\sigma ^{2}\right )$) and participants’ true means are normally distributed around the group-level mean ($\theta _{i} \sim \mathcal {N}\left (\mu ,\tau ^{2}\right )$). This setup acknowledges the fact that participants’ sample means $\bar {x}_{i}$ are uncertain estimates of their true means *𝜃*_*i*_, and correctly distinguishes the sampling variance *σ*^2^/*K* of the participant means from the variance *τ*^2^ of the true means (see Eq. 3).

A researcher might furthermore propose a uniform prior distribution for the group-level mean *p*(*μ*) ∝ 1. For the sake of clarity, we ignore the priors for the variance parameters and assume that the true values are known. A posterior point estimate of each participant’s true mean is then given by the mean of the posterior distribution of the person’s true mean given the participant’s sample mean and group-level mean, $\theta _{i} \mid \mu , \bar {x}_{i}$. For participant *i*, the posterior point estimate is $\hat {\theta }_{i}=\left (\bar {x}_{i} \tau ^{2}+\mu \sigma ^{2}/K\right )/\left (\tau ^{2}+\sigma ^{2}/K\right )$ and the variance of the posterior distribution is (*τ*^2^*σ*^2^/*K*)/ (*τ*^2^ + *σ*^2^/*K*). The posterior estimate of the participant’s true value $\hat {\theta }_{i}$ is the weighted average of the person’s sample mean and the group-level mean, and as the sampling variance *σ*^2^/*K* increases, more weight is given to the group-level mean, thus pulling, or shrinking, the sample mean towards the group-level mean. As a consequence, the variance of the posterior estimates is smaller than the variance of participants’ true means, *τ*^2^, that is, (*τ*^2^*σ*^2^/*K*)/ (*τ*^2^ + *σ*^2^/*K*) ≤ *τ*^2^. This becomes more obvious when both sides of the inequality are multiplied by *K* and (*τ*^2^ + *σ*^2^/*K*), the denominator of the left-hand side: *σ*^2^ ≤ *τ*^2^*K* + *σ*^2^. Therefore, if posterior estimates from a hierarchical Bayesian model are used in a follow-up frequentist analysis, the group-level variance will be systematically underestimated.

### Consequences for five different analysis strategies

In the preceding sections, we discussed the general mechanisms that give rise to biases if either the hierarchical data structure is ignored or a two-step analysis approach is taken. We now turn to a discussion of the consequences for specific analysis strategies that are frequently seen in statistical practice. We will focus on the case of Bayesian and frequentist *t* tests as these constitute some of the most basic analysis methods in researchers’ statistical toolbox. Nevertheless, the same general conclusions apply to more complex analysis methods.

#### Hierarchical Bayesian *t* test

The correct analysis strategy for hierarchical data with two groups of participants is a hierarchical *t* test. Within the Bayesian framework, statistical hypothesis tests are usually based on Bayes factors, which express the relative likelihood of the data under two competing statistical hypotheses $\mathcal {H}_{0}$ and $\mathcal {H}_{1}$ (Rouder et al., 2009). To compute a Bayes factor, researchers need to specify their prior beliefs about the model parameters they expect to see under each of the competing hypotheses. One particularly convenient way to specify these prior distributions is to express ones expectations about effect size *δ* = (*μ*_2_ − *μ*_1_)/*τ*, where *μ*_*g*_ is the mean of experimental group *g* = 1,2 and *τ* is the group-level standard deviation as above. For the present work, we specified the null hypothesis to be the point null *δ* = 0 and the alternative hypothesis that *δ*≠ 0, which we expressed as a standard normal prior $p(\delta ) = \mathcal {N}(0,1)$. The Bayes factor can then be computed as:

$$\text{BF}_{10} = \frac{p(\textbf{x}\mid \mathcal{H}_{1})}{p(\textbf{x}\mid \mathcal{H}_{0})} = \frac{\int\limits_{{\Theta}}\int\limits_{\delta}p(\textbf{x}\mid\theta,\delta)p(\theta)p(\delta) \mathrm{d}\delta \mathrm{d}\theta}{\int\limits_{{\Theta}}p(\textbf{x}\mid\theta,\delta= 0)p(\theta) \mathrm{d}\theta}, $$

where Θ is the set of model parameters^2^ other than *δ* and **x** is the vector of all measurements *x*_*gij*_ across groups *g*, participants *i*, and repeated measurements *j*. One convenient way to obtain the Bayes factor is known as the Savage–Dickey density ratio (Dickey & Lientz, 1970; Wagenmakers et al., 2010). This method expresses the Bayes factor as the ratio of the prior and posterior densities under the alternative hypothesis at the point null. Specifically, because our null hypothesis is *δ* = 0, the Bayes factor is $\text {BF}_{10} = p(\delta = 0\mid \mathcal {H}_{1}) / p(\delta = 0\mid \textbf {x},\mathcal {H}_{1})$, the prior density at *δ* = 0 divided by the posterior density at *δ* = 0.

One important result of our technical discussion above is that researchers need to specify a hierarchical model that correctly represents the hierarchical structure of their data. In the case discussed here, the model needs to include a trial-level on which repeated measurements for each participant are nested within that person. Moreover, the model needs to include a participant-level on which each participant’s mean is nested within the experimental group. Finally, the model also needs to include a group-level that contains the two experimental groups. Such a model specification guarantees that the different sources of variability in the data, namely the variability of the repeated measurements within each participant, and the variability of the means between participants, are correctly accounted for.^3^ The resulting estimates of the population means and variance will be approximately correct, yielding estimates of the effect size *δ* that lie neither inappropriately close nor inappropriately far from *δ* = 0; hence Bayes factors will correctly represent the evidence for the null and alternative hypothesis.

#### Non-hierarchical Bayesian *t* test

In our discussion above, we showed that modeling participants’ sample means rather than the single trial data (ignoring the hierarchy), ignores the variability of the repeated measurements within each participant and results in an overestimation of the group-level variance *τ*^2^. Such overestimation of the group-level variance will result in effect size estimates *δ* that are too close to 0. Because, given our choice for the prior on *δ*, data associated with small *δ* are more plausible under the null hypothesis of no group difference. Hence Bayes factors based on a non-hierarchical model will unduly favor the null hypothesis when the true effect is *δ*≠ 0. Note that the strength of this bias depends on the choice of the prior distribution for *δ*. A non-local alternative prior, for example, has 0 mass at *δ* = 0 (Johnson & Rossell, 2010), and will therefore result in a much stronger bias towards the null hypothesis if the true effect is *δ*≠ 0.

#### Hierarchical frequentist *t* test

Statistical hypothesis tests within the frequentist framework are based on test statistics that express the ratio of variance accounted for by the experimental manipulation to the standard error of the group-level difference. In the case of a two-sample *t* test for the null hypothesis that there are no group differences, this is simply

$$\begin{array}{@{}rcl@{}} \mathrm{t}=\frac{\hat{\mu}_{2}-\hat{\mu}_{1}}{\hat{\sigma}_{m}} \text{, and}\\ \hat{\sigma}_{m} = \sqrt{\left( \hat{\tau}^{2}_{1}+\hat{\tau}^{2}_{2}\right)/N}, \end{array} $$

where $\hat {\mu }_{1}$ and $\hat {\mu }_{2}$, and $\hat {\tau }^{2}_{1}$ and $\hat {\tau }^{2}_{2}$ are the sample means and variances, respectively, and $\hat {\sigma }_{m}$ is an estimate of the standard error of the group-level difference.

A proper hierarchical analysis constitutes the recommended solution within the frequentist framework (Baayen et al., 2008; Pinheiro & Bates, 2000). However, such a hierarchical analysis might, for some reason, not be feasible. One scenario frequently encountered in practice is a hierarchical Bayesian implementation of a cognitive model for which an equivalent hierarchical frequentist implementation has not been developed (e.g., Matzke et al., 2013, 2015; Ravenzwaaij et al., in press; Wiecki et al.,, 2013; Steingroever et al.,, 2014). In this case, researchers might decide to use the group-level estimates for *μ*_1_,*μ*_2_, and *τ*^2^ from a hierarchical Bayesian model as the basis for their *t* test. Although this strategy is not yet widespread in practice, we include it in our theoretical analysis and in our simulations as a possible alternative to the common but suboptimal strategies of a non-hierarchical or a two-step frequentist *t* test.

Using group-level estimates from a hierarchical Bayesian model in a follow-up frequentist *t* test leads to smaller biases than a non-hierarchical or a two-step frequentist *t* test. Specifically, estimates of the group-level mean in a hierarchical Bayesian model are subject to shrinkage towards the prior mean. However, the degree of shrinkage for the group-level means is mild compared to the shrinkage for participant-level means. Moreover, estimates of the group-level variance obtained from correctly specified hierarchical models will usually not over- or underestimate the true group-level variance. Therefore, *t* tests that are based on such hierarchical Bayesian group-level estimates will tend to be somewhat conservative but will be less biased overall than *t* tests in a two-step or non-hierarchical approach.

#### Non-hierarchical frequentist *t* test

As mentioned before, neglecting the trial-level and basing the analysis on participant means instead (ignoring the hierarchy) leads to an overestimation of the group-level variance. Overestimation of the group-level variance will in turn result in underestimation of *t* values and will bias frequentist *t* tests against the alternative hypothesis.

#### Two-step frequentist *t* test

Our theoretical considerations above showed that hierarchical Bayesian estimates of participants’ means can be strongly affected by shrinkage. Because all estimates are pulled towards a common value, the prior mean, the variance of the estimates can be considerably smaller than the true group-level variance. Therefore, if researchers obtain estimates of participants’ means from a hierarchical Bayesian model and subsequently use these estimates in a frequentist test (two-step approach), the group-level variance will be underestimated, resulting in overestimation of *t* values and a bias in favor of the alternative hypothesis.

### Interim conclusion

To sum up, theoretical considerations indicate that ignoring the hierarchical data structure will lead to an overestimation of the group-level variance. Such an overestimation will bias frequentist as well as Bayesian *t* tests towards the null hypothesis. Taking a two-step analysis approach, on the other hand, will lead to an underestimation of the group-level variance. Consequently, *t* values will be overestimated and tests will be biased towards the alternative hypothesis.

## Part II: Practical ramifications

The theoretical considerations in the previous section indicate that analysis strategies for hierarchical data that ignore the hierarchy or take a two-step approach result in biased statistical tests. To gauge the severity of these biases, we performed a Monte Carlo simulation study using the five analysis strategies discussed above. For the sake of simplicity and comparability with our theoretical results, we focused on a hierarchical data structure with two levels and normal distributions on both levels. Nevertheless, the overall patterns observed here apply to more complex cases with different distributions or numbers of hierarchical levels.

### Constructing a data-generating model

To simulate a realistic experimental setup, we considered a typical psychological experiment in which the goal is to assess the effect of an experimental manipulation on a variable of interest, say RT. To this end, participants are randomly assigned to one of two experimental conditions. Subsequently, each participant’s RT is measured repeatedly.

**Fig. 1:**
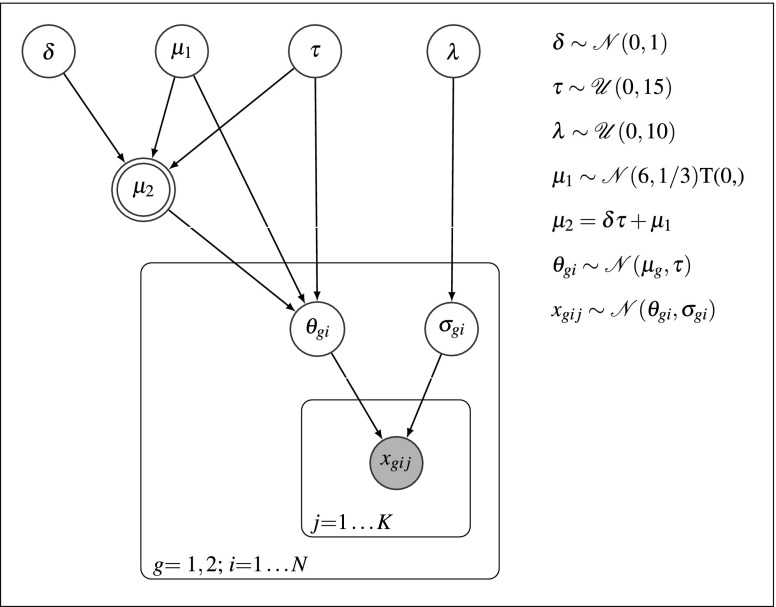
Full hierarchical model. $\mathcal {N}$ denotes the normal prior, $\mathcal {U}$ denotes the uniform prior, and T(0,) indicates truncation at 0

A hierarchical Bayesian model of such an experiment is shown in Fig. 1. On the first, trial-level, the model assumes that repeated measurements *x*_*gij*_ for participant *i* in group *g* (shaded, observed node in the innermost plate) are drawn from a normal distribution with mean *𝜃*_*gi*_ and variance $\sigma ^{2}_{gi}$ (unshaded, stochastic nodes in the intermediate plate). On the second, participant-level, the mean *𝜃*_*gi*_ for each participant is drawn from a normal distribution with mean *μ*_*g*_ (for *μ*_1_, second unshaded, stochastic node from the left at the top; for *μ*_2_ double-bordered, deterministic node in the outer plate; the node is shown as deterministic because *μ*_2_ is fully determined by *δ*, *τ*, and *μ*_1_) and standard deviation *τ* (third unshaded, stochastic node from the left at the top). The participant-specific sampling variance $\sigma ^{2}_{gi}$ is drawn from a half-normal distribution with mean 0 and standard deviation *λ* (fourth unshaded, stochastic node from the left at the top; see Gelman, 2006; Chung et al., 2013, for a discussion of choices for prior distributions for variance parameters). We further assumed that only the group mean, *μ*_*g*_, differs between groups by *δτ*, where *δ* (leftmost unshaded, stochastic node at the top) is the standardized effect size (i.e., we assumed equal variances across groups; *μ*_2_ = *μ*_1_ + *δτ*).

As we had little prior information regarding plausible parameter values for the hierarchical model yet a wealth of data to constrain the posterior estimates of the parameter values, we followed Edwards et al.,’s (1963) principle of stable estimation. That is, for the group-level model parameters *μ*_1_, *τ*, and *λ*, for which there was no default prior distribution available, we specified the prior to be relatively uninformative across the range of values supported by the data. Therefore, we assigned the group-level mean *μ*_1_ a positive-only (truncated) normal distribution^4^ with mean 6 and standard deviation 1/3; we assigned the standard deviation *τ* of participants’ true values *𝜃*_*i*_ a uniform distribution ranging from 0 to 15 (Gelman, 2006); we assigned the standard deviation *λ* of the distribution of sampling variances a uniform prior ranging from 0 to 10. Exploratory analyses using different distributions for *τ* and *λ* yielded similar results.

We implemented our model in Stan Development Team (2016a, 2016b) and ran MCMC chains until convergence (Gelman-Rubin diagnostic $\hat {R} \leq 1.05$; Gelman and Rubin, 1992). We obtained 20,000 samples from three chains for each model parameter, of which we discarded 2000 samples as burn-in. Thinning removed a further three out of every four samples, leaving us with a total of 4500 posterior samples per parameter and chain. We then used the mean of the posterior samples to parameterize the three group-level parameters ($\hat {\mu }_{1}= 6.52$, $\hat {\tau }= 0.16$, $\hat {\lambda }= 0.29$) of our model. To generate data for our Monte Carlo simulations, we set the fourth group-level parameter, *δ*, to a pre-specified value (see next section), and sampled *N* values of the participant-level parameters (*𝜃*_*gi*_, $\sigma ^{2}_{gi}$), representing simulated participants, for each experimental group. We subsequently sampled *K* values of the trial-level parameter (*x*_*gij*_) for each simulated participant in each experimental group (i.e., a total of 2 × *N* × *K* values).

### Designing the Monte Carlo simulations

We generated data from the hierarchical Bayesian model as described above and applied five different analysis strategies. Repeating this process 200 times for each simulation allowed us to quantify the degree of bias introduced by the different strategies.

We varied three parameters that should influence the degree to which different analysis strategies bias statistical results. The number of simulated trials per participant, *K*, varied over four levels (*K* ∈{2,5,15,30}). The number of simulated participants in each group, *N*, also varied over four levels (*N* ∈{2,5,15,30}). Here the smallest values, *K* = 2 and *N* = 2, were included to illustrate the mechanism of the different statistical biases in extreme cases. We manipulated the size of the effect between groups, *δ*, which was chosen from the set {0,0.1,0.5,1}. In each simulation, we used one combination of parameter values, resulting in a total of 64 simulations with 200 data sets each. The R-code for the simulations is available in the online appendix: osf.io/uz2nq.

### Implementation of analysis strategies

#### Hierarchical Bayesian *t* test

For the hierarchical Bayesian analysis, we fit the complete hierarchical model described in the section “Constructing a data-generating model” (see also Fig. 1) to the simulated data. We assigned the group-level parameters *μ*_1_, *τ*, and *λ* the priors described above. Moreover, we assigned the standardized effect size *δ* a normal prior with mean 0 and standard deviation 1 (Rouder et al., 2009).

To analyze the simulated data, we implemented the hierarchical model in Stan (RStan version 2.9.0; Stan Development Team, 2016a, 2016b) and ran MCMC chains until convergence (Gelman-Rubin diagnostic $\hat {R} \leq 1.05$; Gelman and Rubin, 1992) with the same settings as described above (i.e., we obtained 20,000 samples from three chains, of which 2000 samples were discarded as burn-in and a further three out of every four samples were removed by thinning). We then estimated the Bayes factors using the Savage–Dickey method (Dickey & Lientz, 1970; Wagenmakers et al., 2010) based on logspline density fits of the posterior samples for *δ* (Stone et al., 1997).

#### Non-hierarchical Bayesian *t* test

**Fig. 2:**
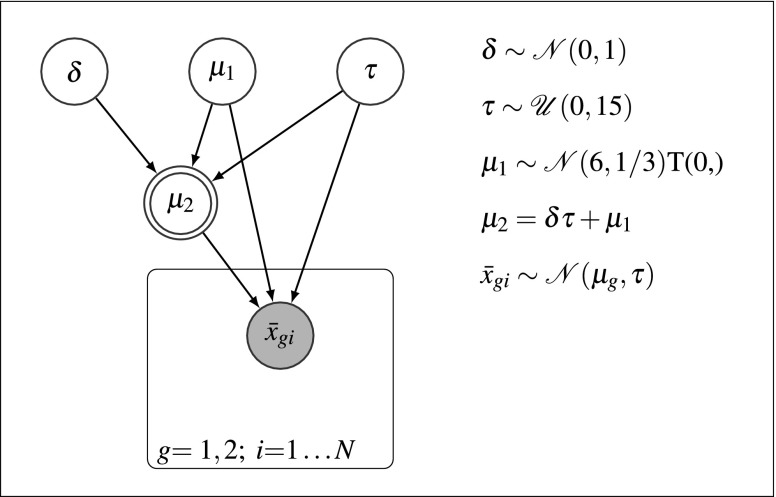
Non-hierarchical model. $\mathcal {N}$ denotes the normal prior distribution, $\mathcal {U}$ denotes the uniform prior, and T(0,) indicates truncation at 0

For the non-hierarchical Bayesian analysis, we considered a model that has the same overall structure as the hierarchical model but ignores the participant-level (Fig. 2). Specifically, the model represents individual participants *i* in group *g* by their participant means $\bar {x}_{gi}$ (shaded, deterministic node in the innermost plate), thus ignoring the sampling variance associated with the participant means. The participant means are in turn drawn from a normal distribution with mean *μ*_*g*_ (for *μ*_1_, second unshaded, stochastic node at the top; for *μ*_2_ double-bordered, deterministic node in the outer plate; the node is shown as deterministic because *μ*_2_ is fully determined by *δ*, *τ*, and *μ*_1_) and standard deviation *τ* (right unshaded, stochastic node at the top). Groups again only differ in their mean *μ*_*g*_ by *δτ*, where *δ* (left unshaded, stochastic node at the top) is the standardized effect size.

We ran MCMC chains for the model until convergence and obtained 5000 samples from three chains for each model parameter, of which we discarded 500 samples as burn-in, leaving a total of 4500 posterior samples per parameter and chain. Thinning was not necessary, as we did not observe any noteworthy autocorrelations. As with the hierarchical model, we estimated Bayes factors using the Savage–Dickey method.

We based the hierarchical frequentist *t* test on group-level estimates from the hierarchical Bayesian model. In particular, we computed the median of the posterior samples for the group-level means *μ*_*g*_ and standard deviation *τ* and used these summary statistics to compute the *t* values. We set the type I error rate for the two-sided test to the conventional *α* = .05.

#### Non-hierarchical frequentist *t* test

We based the non-hierarchical frequentist *t* test on the participant means $\bar {x}_{gi}$. We therefore computed estimates of the group-level means and standard deviation by averaging the participant means in each experimental group and computing the pooled standard deviation of the participant means, respectively. As for the hierarchical *t* test, we set *α* = .05.

#### Two-step frequentist *t* test

For the two-step analysis approach, we used participant-level estimates from the hierarchical Bayesian model as input for a frequentist *t* test. We therefore computed the median of the posterior samples for each participant’s estimated true mean *𝜃*_*gi*_. We then obtained estimates of the group-level means and standard deviation by averaging the posterior medians of the posterior estimates in each experimental group and computing their pooled standard deviation, respectively. As for the hierarchical *t* test, we set *α* = .05.

### Results

To anticipate our main conclusion, our simulation results corroborate the theoretical predictions. Specifically, an analysis that takes the hierarchical structure of the data into account leads to approximately correct inferences, whereas analyses that neglect the hierarchical data structure lead to an overestimation of the group-level variance, and thus bias Bayesian and frequentist *t* tests towards the null hypothesis of no group difference. Moreover, taking a two-step analysis approach leads to an underestimation of the group-level variance, and thus biases *t* tests towards the alternative hypothesis. In addition, the simulations also revealed a result that was not obvious from the theoretical analyses; this result will be discussed in more detail below.

Below we will focus on only the most extreme cases (*N* ∈{2, 30}, *K* ∈{2, 30}, *δ* ∈{0,1}), as they provide the clearest illustration of the consequences of the different analysis strategies. Nevertheless, the results presented here hold generally. The results of the full set of simulations can be found in the online appendix: osf.io/uz2nq.

#### Hierarchical Bayesian *t* test

**Fig. 3:**
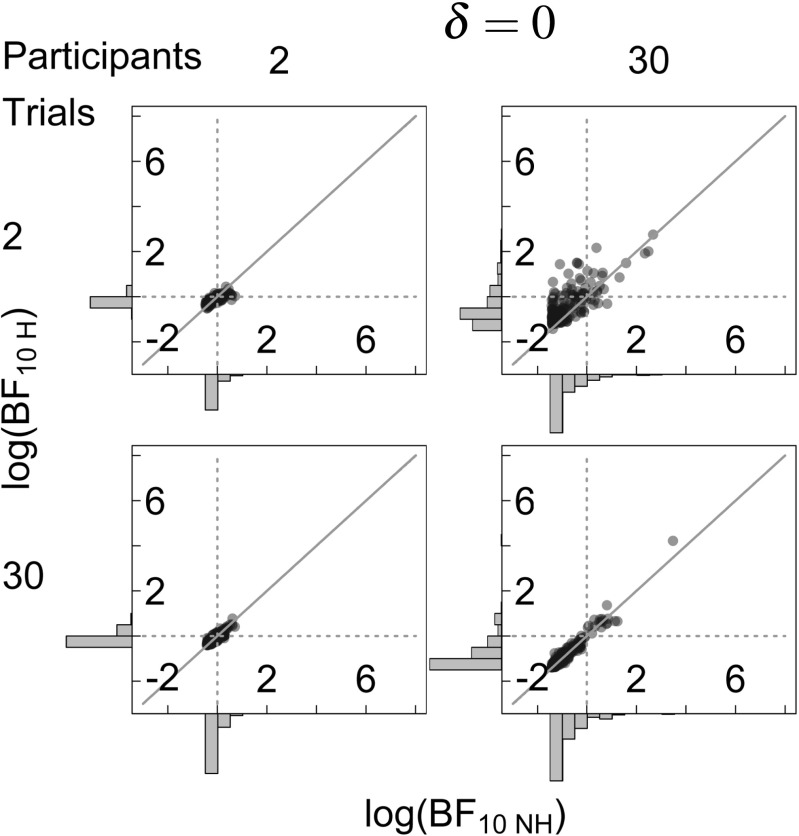
Outcomes of the Bayesian analysis under the hierarchical and non-hierarchical Bayesian model for different numbers of simulated trials (K) and participants (N) for *δ* = 0. The scatterplot shows a comparison of log-Bayes factors for the hierarchical (BF_10*H*_, *y*-axis) and non-hierarchical (BF_10*NH*_, *x*-axis) Bayesian model. The gray diagonal line shows where log-Bayes factors should fall in the case of equality ($\log {\text {BF}_{10 H}}=\log {\text {BF}_{10 NH}}$). The gray dotted lines indicate the indecision point where $\log {\text {BF}}= 1$. *Histograms* show the marginal distribution of the log-Bayes factors

Figure 3 shows a comparison of the hierarchical and the non-hierarchical Bayesian *t* test for *δ* = 0. Data points are the natural logarithm of the Bayes factors under the hierarchical and non-hierarchical model (scatter plots), which means that values below 0 indicate evidence for the null hypothesis whereas values above 0 indicate evidence for the alternative hypothesis; marginal distributions of the Bayes factors under each model are shown on the sides. Panels give the results for different numbers of trials (K) and participants per group (N). The horizontal dashed line indicates the point where hierarchical log-Bayes factors are 0 and favor neither the null nor the alternative hypothesis.

The *y*-axis shows the hierarchical log-Bayes factors for 200 simulations. Hierarchical Bayes factors constitute the correct Bayesian analysis of the simulated data. When the number of participants is low, these Bayes factors are largely unaffected by the number of trials per participant (compare top and bottom row in the left column) and log-Bayes factors cluster around 0, which indicates a lack of evidence. However, when the number of participants is large, Bayes factors become smaller as the number of trials per participant increases, thus increasingly favoring the null hypothesis that there is no difference between groups (compare top and bottom row in the right column).

**Fig. 4:**
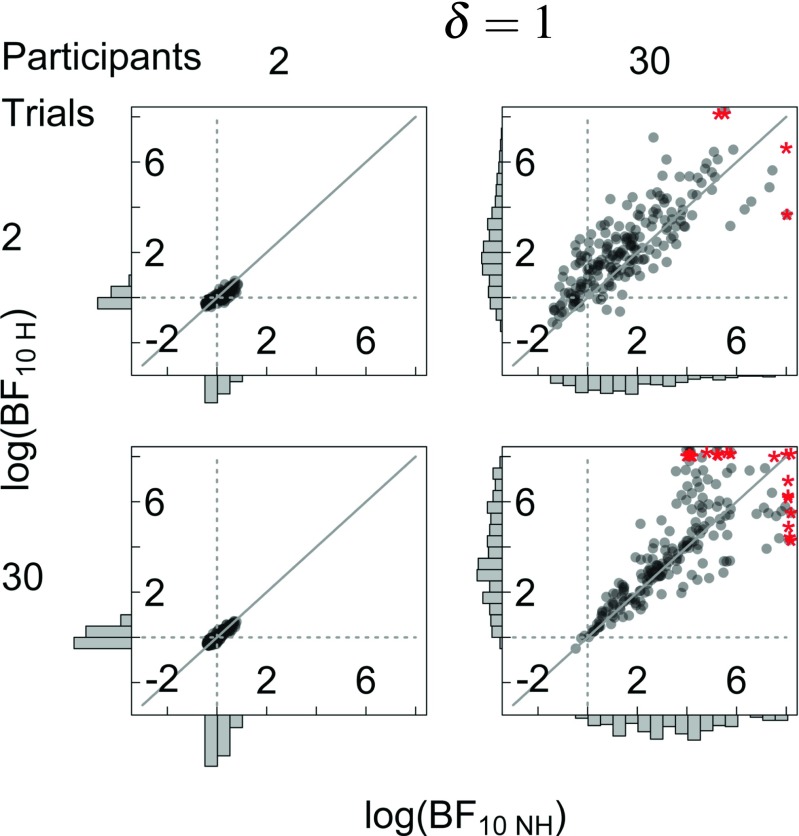
Outcomes of the Bayesian analysis under the hierarchical and non-hierarchical Bayesian model for different numbers of simulated trials (K) and participants (N) for *δ* = 1. The scatterplot shows a comparison of log-Bayes factors for the hierarchical (BF_10*H*_, *y*-axis) and non-hierarchical (BF_10*NH*_, *x*-axis) Bayesian model. Red asterisks indicate outliers (outliers are jittered to prevent visual overlap). The gray diagonal line shows where log-Bayes factors should fall in the case of equality ($\log {\text {BF}_{10 H}}=\log {\text {BF}_{10 NH}}$). The gray dotted lines indicate the indecision point where $\log {\text {BF}}= 1$. *Histograms* show the marginal distribution of the log-Bayes factors

Figure 4 shows the comparison of the hierarchical and the non-hierarchical Bayesian *t* test for *δ* = 1. The results are complementary to the results for *δ* = 0; hierarchical Bayes factors, shown on the *y*-axis, cluster around 0 when the number of participants is low, irrespective of the number of trials per participant (compare top and bottom row in the left column). This indicates a lack of evidence. On the other hand, when the number of participants is large, hierarchical Bayes factors become larger as the number of trials per participant increases (compare top and bottom row in the right column), thus increasingly favoring the alternative hypothesis (compare top and bottom row in the right column).

#### Non-hierarchical Bayesian *t* test

The non-hierarchical log-Bayes factors for *δ* = 0 are shown on the *x*-axis in Fig. 3, the vertical dashed line indicates the point where the log-Bayes factors are 0. Similar to the hierarchical Bayes factors, when the number of participants is low, non-hierarchical log-Bayes factors are unaffected by the number of trials per participant and cluster around 0, which indicates a lack of evidence (compare top and bottom row in the left column). However, when the number of participants is large, Bayes factors become smaller as the number of trials per participant increases, thus increasingly favoring the null hypothesis (compare top and bottom row in the right column).

The non-hierarchical Bayes factors for *δ* = 1, shown on the *x*-axis in Fig. 4. These Bayes factors cluster around 0 when the number of participants is low, irrespective of the number of trials per participant (compare top and bottom row in the left column). This indicates a lack of evidence. On the other hand, when the number of participants is large, non-hierarchical Bayes factors become larger as the number of trials per participant increases, thus increasingly favoring the alternative hypothesis (compare top and bottom row in the right column).

Importantly, in the top right scatter plots of Figs. 3 and 4, most data points lie above the diagonal. This indicates that when the number of participants is large and the number of trials per participant is low, non-hierarchical Bayes factors are biased towards the null hypothesis. However, when the number of trials per participant is large, this bias disappears (compare bottom right panels in Figs. 3 and 4).

**Fig. 5:**
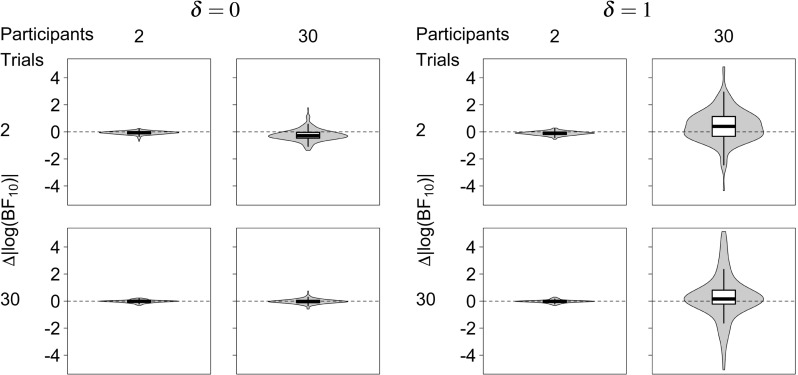
Differences between log-Bayes factors under the hierarchical and non-hierarchical Bayesian model. Violin plots show the distribution of differences between absolute log-Bayes factors, $| \log {\text {BF}_{10 H}} | - | \log {\text {BF}_{10 NH}} |$, for different numbers of simulated trials (K) and participants (N). Dashed horizontal lines indicate no difference in log-Bayes factors

Similar patterns can be seen in Fig. 5, which shows the differences in absolute log-Bayes factors under the hierarchical and the non-hierarchical model. Dashed gray lines show the point where Bayes factors under both models are equal. The results for *δ* = 0, shown on the left, indicate that in most situations considered here hierarchical and non-hierarchical Bayes factors are approximately equal. However, when the number of participants is large and the number of trials per participant is relatively small (top right panel), differences between absolute log-Bayes factors are smaller than 0, which means that absolute non-hierarchical Bayes factors are larger than absolute hierarchical Bayes factors, and thus tend to overstate the evidence for the null hypothesis. The results for *δ* = 1, shown on the right, again indicate that in most situations considered here hierarchical and non-hierarchical Bayes factors are approximately equal. However, when the number of participants is large and the number of trials per participant is relatively small (top right panel), differences between absolute log-Bayes factors are larger than 0, which means that non-hierarchical Bayes factors are smaller than hierarchical Bayes factors, and thus are biased towards the null hypothesis.

**Fig. 6:**
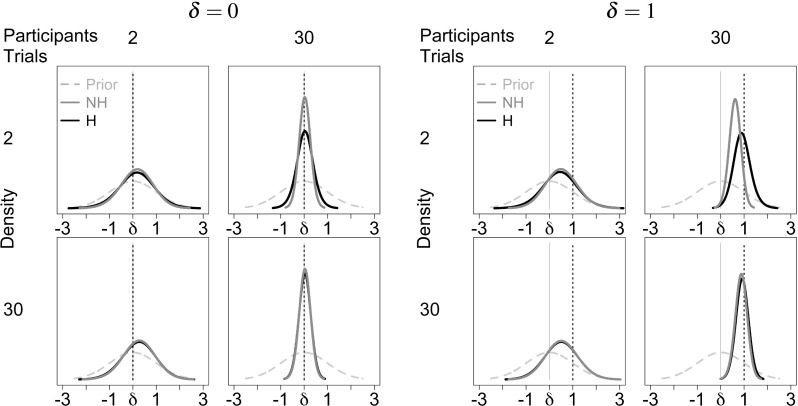
Posterior distribution of effect size *δ* under the hierarchical and non-hierarchical Bayesian model for different numbers of simulated trials (K) and participants (N). Distributions shown are the prior (light gray dashed lines) and quantile-averaged posterior distributions of *δ* under the hierarchical (H, black) and non-hierarchical model (NH, dark gray) for *δ* = 0 (left subplot) and *δ* = 1 (right subplot). The gray solid vertical line indicates the mean of the prior distribution and the black dashed vertical line shows the true value of *δ*

The above observations can be accounted for by examining the behavior of the posterior distributions on which the Bayes factors are based. Figure 6 shows the prior and quantile-averaged posterior distributions for *δ* under the hierarchical and the non-hierarchical model. Panels show the results for different numbers of trials (K) and participants per group (N) for *δ* = 0 (left subplot) and *δ* = 1 (right subplot). The posterior distributions under the hierarchical and the non-hierarchical model are very similar under most conditions except when the number of participants is large and the number of trials per participant is small (top right panel in both subplots). When *δ* = 0, the modes of the posterior distributions are equal under both models (top right panel in the left subplot), whereas when *δ* = 1, the mode under the non-hierarchical model is systematically smaller than the mode under the hierarchical model (top right panel in the right subplot). This pattern is due to the fact that the non-hierarchical model ignores the sampling variance associated with participant means, which leads to an overestimation of the group-level variance and thus biases the posterior distribution of the effect size towards the null hypothesis *δ* = 0 when the true effect is *δ* = 1.

The quantile-averaged posteriors in Fig. 6 furthermore reveal a subtle overconfidence bias in the non-hierarchical model. When the number of participants is large and the number of trials per participant is small (top right panel in both subplots), the posterior under the non-hierarchical model is more peaked than under the hierarchical model, which means that the non-hierarchical model overstates the confidence that can be placed in estimates of the effect size *δ*. Although we did not anticipate this result from our theoretical analysis, the overconfidence bias is nevertheless in line with our theoretical considerations. Because the non-hierarchical model ignores the sampling variance associated with participant means as a separate source of uncertainty about *δ*, the posterior variance of *δ* is underestimated.

The consequences of the behavior of the posteriors for Bayes factors are straightforward. First consider *δ* = 0, where the modes of the posterior distribution under both models are equal but, due to the overconfidence bias, the posterior under the non-hierarchical model is more peaked. This means that the non-hierarchical posterior has higher density at *δ* = 0, resulting in Bayes factors that provide stronger support for the null hypothesis than hierarchical Bayes factors. Second, consider *δ* = 1. In this case, due to the overconfidence bias, the posterior under the non-hierarchical model is again more peaked. This means that if the posterior modes under both models were similar, the non-hierarchical model would yield larger Bayes factors than the hierarchical model. However, for the simulations reported here, the mode of the non-hierarchical posterior lies considerably closer to *δ* = 0 than the mode of the hierarchical posterior, which mitigates the effect of the lower posterior standard deviation and leads to a bias towards the null hypothesis. Nevertheless, the trade-off between the two biases is subtle and differences in the posterior mode are not guaranteed to fully offset differences in posterior standard deviation between the hierarchical and the non-hierarchical model. Smaller differences between the number of participants and the number of trials per participant than reported here, for example, can result in non-hierarchical Bayesian *t* tests that overstate the evidence for the alternative hypothesis compared to hierarchical Bayesian *t* tests (see Figures A2-A4 and A6-A8 in the online appendix: osf.io/uz2nq, for examples).

#### True values

**Fig. 7:**
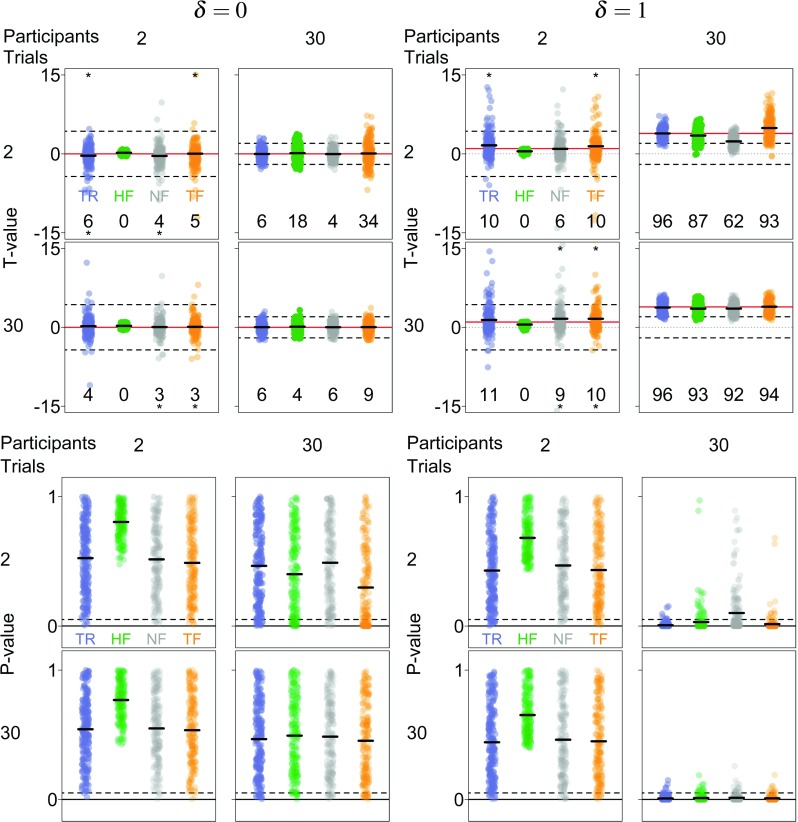
Outcomes of the frequentist analysis for different numbers of simulated trials (K) and participants (N). Top row: t values for *δ* = 0 (left subplot) and *δ* = 1 (right subplot). Dotted lines show *t* = 0, dashed lines show the critical *t* value in a two-sided *t* test with *α* = .05, and red lines show the theoretical *t* value. Dots are true *t* values (TR; blue), *t* values from a hierarchical frequentist strategy (HF; green), non-hierarchical frequentist strategy (NF; grey), and two-step frequentist strategy (TF; orange); asterisks denote outliers (outliers are jittered to prevent visual overlap). Numbers at the bottom indicate the proportion of significant *t* values (out of 200 *t* tests). Bottom row: *p* values for *δ* = 0 (left subplot) and for *δ* = 1 (right subplot). Solid lines indicate *p* = .05. Dots are true *p* values (blue), *p* values from a hierarchical frequentist strategy (green), non-hierarchical strategy (grey), and two-step frequentist strategy (orange). Data points are jittered for improved visibility

To obtain a standard for our comparisons between the three frequentist analysis strategies, we computed the true *t* values and *p* values for each simulated data set based on the true participant means, which are usually not available to researchers in empirical data sets. Figure 7 shows the true *t* values (top rows) and *p* values (bottom rows) and the *t* and *p* values obtained by each of the three frequentist analysis strategies for different numbers of trials (K) and participants per group (N) for *δ* = 0 (left column) and *δ* = 1 (right column). Short thick black lines indicate the mean *t* values and *p* values across the 200 simulations. Numbers at the bottom of each panel show the proportion of significant *t* values.

The true *t* values (TR, blue) are sensitive to the number of participants in each experimental group. When *δ* = 0, the values are symmetrically distributed around 0 and cluster more closely together for larger numbers of participants (compare blue dots in the left and right panels of the top left subplot). The type I error rate approximately equals the nominal *α* = .05. The corresponding *p* values (bottom left subplot) are uniformly distributed over the range from 0 to 1, as is expected if the null hypothesis is true. When *δ* = 1, the *t* values are symmetrically distributed around the theoretical value and cluster more closely together for larger numbers of participants (compare left and right panels of the top right subplot). The corresponding *p* values rapidly approach 0 as the number of participants increases (bottom right subplot).

#### Hierarchical frequentist *t* test

When *δ* = 0, *t* values that are based on group-level estimates from a hierarchical Bayesian model (HF, green) tend to cluster more closely around 0 than the true *t* values for small numbers of participants (compare green to blue dots in the left panels of the top left subplot). However, when the number of participants is large, the *t* values are as variable as the true *t* values (right panels in the top left subplot). This is also reflected in the observed type I error rate that is far below the nominal *α* = .05 when there are few participants but, somewhat unexpectedly, surpasses that theoretical value for large numbers of participants and small numbers of trials. The corresponding *p* values cluster near 1 for small numbers of participants (left panels in the bottom left subplot) but become more evenly spread over the range from 0 to 1 for large numbers of participants, especially when the number of trials per participant is relatively large (right panels in the bottom left subplot). When *δ* = 1, the *t* values are, on average, smaller than the true *t* values (top right subplot), except when the number of participants and the number of trials per participant are large; the power of hierarchical *t* tests lags behind that for *t* tests based on the true *t* values. The *p* values cluster near 1 for small numbers of participants (left panels in the bottom right subplot) but approach 0 as the number of participants increases, especially when the number of trials per participant is large (right panels in the bottom right subplot).

These results can be understood by considering the behavior of the group-level hierarchical Bayesian estimates used in the frequentist analysis. Specifically, because the hierarchical Bayesian model takes the hierarchical structure of the data into account, estimates of the group-level variance *τ* are not overly biased. The posterior estimate of each group-level mean *μ*_*g*_ is the weighted average of the prior mean and participants’ sample means. For small numbers of participants, this posterior estimate is shrunken towards the prior mean but as the number of participants increases, the posterior estimate increasingly depends on participants’ sample means. Consequently, when the number of participants is small, *t* values tend to be underestimated whereas when the number of participants is large, this underestimation disappears.

#### Non-hierarchical frequentist *t* test

When *δ* = 0, *t* values that are based on participant means (NF, gray) are similarly distributed as the true *t* values (compare grey to blue dots in the top left subplot) and the observed type I error rate is roughly in keeping with the nominal *α* = .05. The corresponding *p* values uniformly span the range from 0 to 1 (bottom left subplot). However, when *δ* = 1 and the number of participants is large but the number of trials per participant is small, the *t* values are systematically smaller than the true values (top right subplot), and power consequently lags behind the power associated with the true *t* values. This pattern is also reflected in the *p* values, which approach 0 more slowly than the true *p* values (bottom right subplot).

These results are accounted for by the fact that basing *t* values on participant’s sample means $\bar {x}_{gi}$ ignores the sampling variance associated with those means. Consequently, the group-level variance is overestimated, which leads to an underestimation of *t* values.

#### Two-step frequentist *t* test

When *δ* = 0, *t* values that are based on participant-level estimates from a hierarchical Bayesian model (TF, orange) are in most cases similar to the true *t* values (top left subplot). However, when the number of participants is large and the number of trials per participant is small, *t* values from a two-step analysis are more variable than the true *t* values (compare orange and blue dots in the top right panel of the top left subplot) and the type I error rate is up to six times the nominal *α* = .05. The *p* values show a corresponding pattern (bottom left subplot), being uniformly distributed between 0 and 1 except when the number of participants is large and the number of trials per participants is small, in which case the *p* values rapidly approach 0 (top right panel in the bottom left subplot). When *δ* = 1, *t* values from a two-step analysis are again largely similar to the true *t* values (top right subplot). However, when the number of participants is large and the number of trials per participant is small, *t* values from a two-step analysis are larger and more variable than the true *t* values (compare orange and blue dots in the top right panel of the top right subplot). Nevertheless, the power of two-step *t* tests differs only slightly from that of *t* tests based on the true *t* values. The corresponding *p* values show a complementary pattern (bottom right subplot), being relatively uniformly distributed between 0 and 1 when the number of participants is small but rapidly approaching 0 when the number of participants is large (top right panel in the bottom left subplot).

These results are again easily explained by the Bayesian estimators based on which the *t* values were computed. Participant-level estimates from a hierarchical Bayesian model are shrunken towards a common value, the prior mean, and shrinkage is strongest when the number of participants is large and the number of trials per participant is small. Therefore, in these situations, the group-level variance is underestimated, resulting in an overestimation of *t* values.

#### Interim conclusion

The results of our simulation study corroborate the theoretical predictions. Bayesian and frequentist *t* tests that ignore the hierarchical data structure are biased in favor of the null hypothesis that there is no difference between groups. Frequentist *t* tests in a two-step approach tend to unduly favor the alternative hypothesis. In addition, our simulations revealed an overconfidence bias in non-hierarchical Bayesian *t* tests, which tend to overstate the support for the hypothesis the Bayes factor favors. This overconfidence bias, which we did not anticipate in our theoretical analysis, is explained by the nature of the posterior distributions, which are too peaked when the hierarchical data structure is ignored.

## Discussion

Over the last decade, the use of cognitive models in the analysis of experimental data has become increasingly popular in cognitive science, a trend that has been further reinforced by the recent popularization of hierarchical Bayesian implementations of cognitive models (Rouder & Jun, 2005; Rouder et al., 2003). This development has had many positive effects, such as facilitating experimental studies based on quantitative predictions and offering new ways of connecting neurophysiological and psychological theories of the human mind (Forstmann et al., 2011). However, the increased use of cognitive models comes at the cost of an increased number of suboptimal applications of cognitive models.

In the present study, we set out to demonstrate how faulty analysis strategies in cognitive modeling of hierarchical data can lead to biased statistical conclusions. We considered two inappropriate approaches, namely ignoring the hierarchical data structure and taking a two-step analysis approach. Both of these approaches are highly prevalent in recent studies and might therefore introduce substantial biases into the literature. Well-established theoretical results predict that ignoring the hierarchy leads to an overestimation of the group-level variance, which should result in a bias towards the null hypothesis (see also Box and Tiao, 1992). Taking a two-step approach, on the other hand, should lead to an underestimation of the group-level variance, which should result in a bias towards the alternative hypothesis. To illustrate the severity of these biases, we conducted a Monte Carlo study in which we generated data for a two-group experiment. For illustrative purposes, we considered a simple statistical model with normal distributions on the group level and on the participant level. For the Bayesian analysis of the data, we computed Bayes factors for the effect size based on either a hierarchical or a non-hierarchical model. In line with our predictions, the simulations showed that non-hierarchical Bayes factors exhibited a bias towards the null hypothesis. In addition, the simulations also revealed an overconfidence bias in non-hierarchical Bayes factors, which overstate the strength of the evidence provided by the data. Although we did not anticipate this result from our theoretical analysis, the overconfidence bias is explained by the theoretical properties of the posterior distributions on which the Bayes factors are based. Both tendencies, the bias towards the null hypothesis and the overconfidence bias, were most pronounced when the number of simulated trials was small and the number of participants was large.

For the frequentist analysis, we computed *t* tests that were either based on participants’ sample means, which ignore the hierarchical data structure, or participant-level posterior estimates from a hierarchical Bayesian model that represent a two-step approach. In addition, we computed frequentist *t* tests that were based on group-level posterior estimates from a hierarchical Bayesian model. Because the group-level posterior estimates respect the hierarchical data structure, we expected that this analysis strategy might mitigate the biases of a two-step approach. Our results were again largely in line with previous theoretical results. *t* tests based on participants’ sample means resulted in an underestimation of *t* values and a loss of power; these biases were particularly strong when the number of participants was large and the number of trials was small. *t* tests based on hierarchical Bayesian participant-level estimates resulted in highly variable *t* values, leading to considerable type I error inflation, especially when the number of participants was large and the number of trials was small. *t* tests based on hierarchical Bayesian group-level estimates, on the other hand, resulted in *t* values that were biased towards the null hypothesis, especially when the number of participants was large and the number of trials per participant was relatively low.

Taken together, our results show that ignoring the hierarchical data structure or taking a two-step analysis approach can bias researchers’ conclusions. These biases are most pronounced when only little data is available for each participant and the number of participants is large. Under these circumstances, the sampling variance will be greatest and, consequently, the group-level variance, if not modeled correctly, will be overestimated to the highest degree, thus also maximizing shrinkage in Bayesian parameter estimates.

### Ramifications for cognitive modeling

The simulations presented here used a simple statistical model for illustrative purposes. This raises the question of how our results generalize to hierarchical cognitive models. To answer this question, we first note that, although the strength of each type of bias will depend on the particular cognitive model, the general mathematical mechanisms discussed in the first part of this paper will hold for any cognitive model. Ignoring the variance on one hierarchical level in a model will lead to a propagation of the variance to another hierarchical level, and will therefore bias statistical tests irrespective of the particular model. Similarly, shrinkage will reduce the variance of the posterior estimates in any hierarchical Bayesian model, and hence bias statistical tests irrespective of the particular model.

One important difference between most cognitive models and the statistical model considered here is that the parameters in cognitive models are often highly correlated. In the diffusion decision model, for example, correlations between parameters tend to range from 0.5 upward (Ratcliff & Tuerlinckx, 2002) and misestimation of some parameters can critically affect estimation performance for other parameters (Boehm et al., 2018). Consequently, biases introduced in one parameter by inappropriate analysis strategies might carry over to other parameters, and thus affect statistical tests across model parameters. Although researchers might only be interested in testing the effect of an experimental manipulation on a particular model parameter, inappropriately modeling the hierarchical data structure on another parameter can still affect tests on the parameter of interest. It therefore seems that correctly accounting for the hierarchical data structure is even more important in cognitive models than in the simple statistical model considered here.

Despite the theoretical advantages that hierarchical cognitive models have to offer, implementing the full hierarchical structure for all model parameters might not always be feasible or even desirable. Hierarchical cognitive models are notoriously difficult to fit (e.g., Turner et al., 2013) and researchers might not have sufficient data to estimate a full random-effects structure on all model parameters. Moreover, fitting a fully hierarchical model might come at the expense of decreased statistical power (Matuschek et al., 2017). One possible solution might be to implement a fully hierarchical structure only for some model parameters. However, this strategy comes with two complications. First, different model selection criteria build on different definitions of what constitutes the ‘best’ model, and might therefore select different models for the same data (Aho et al., 2014; McQuarrie & Tsai, 1998). Second, model selection is always associated with uncertainty (Jeffreys, 1961; Silberzahn & Uhlmann, 2015), hence selecting a single model carries the risk of missing relevant random effects. One possible solution is provided by multimodel inference. Bayesian model averaging, for example, avoids the need to select a single model and accounts for model uncertainty by weighing the results of each model by the plausibility of the model in light of the data (Gronau et al., 2017; Hoeting et al., 1999). Similarly, model averaging can also be performed in a frequentist setting, for instance using Akaike weights (Burnham & Anderson, 2002).

Statistically sound applications of hierarchical Bayesian cognitive models are further hampered by the inflexibility of existing software packages. For example, the use of hierarchical Bayesian cognitive models has been strongly advocated for clinical applications, where these methods help address the strong constraints on data collection (Matzke et al., 2013, in press; Shankle et al., 2013; Wiecki et al., 2013); by pooling all available information, hierarchical Bayesian models provide more reliable parameter estimates than if each participant’s data were modeled individually. However, as our simulation study demonstrates, using hierarchical Bayesian participant-level parameter estimates in ANOVA-type analyses can lead to a substantial type I error inflation. A more appropriate analysis strategy would be to include the clinical variables of interest in the hierarchical Bayesian model itself. Unfortunately, while some software packages already come equipped with a basic capability for modeling covariates (e.g., HDDM; Wiecki et al., 2013), or can be easily extended with a general linear model (Boehm et al., in press), other software packages lack this flexibility. In these cases, users will need to seek other strategies to avoid statistical biases in their analyses. One strategy we explored here was to use group-level parameter estimates from the hierarchical Bayesian model, rather than participants-level estimates, as input for ANOVA-type analyses. Our simulations showed that although the type I error rate inflation caused by this strategy is considerably smaller than that caused by a two-step analysis approach, the type I error rate can still be up to four times the nominal rate. We therefore recommend against the use of group-level estimates from a hierarchical Bayesian model in follow-up statistical tests.

Careful examination of the mechanisms underlying the biases created by a two-step analysis approach suggests further ways to alleviate the problem. As our simulations show, using participant-level posterior estimates in a *t* test leads to an overestimation of *t* values because the group-level variance is underestimated. This overestimation is caused by shrinkage, which pulls less reliable participant-level estimates more strongly towards the group mean. However, while shrinkage corrects the location of the participant-level posteriors, it does not eliminate the posterior variance associated with these estimates. On the other hand, if participant-level point estimates are used to estimate the group-level variance, as is done in a two-step approach, the posterior variance associated with these estimates is ignored and the group-level variance is thus underestimated.

An alternative approach that correctly takes the posterior variance of the participant-level estimates into account is the method of plausible values (Ly et al., in press; Mislevy, 1991; Marsman et al., 2016). In this approach, a single sample is drawn from the posterior distribution of the participant-level parameters, which accounts for the fact that the posterior distributions have a certain variance. The resulting samples are referred to as plausible values and can be used to compute an estimate of the group-level mean and variance. Repeating the sampling process several times will give sets of estimates of the group-level mean and variance that, if pooled correctly (Mislevy, 1991), can be used to compute a *t* value.

Finally, irrespective of the technical explanations for our findings discussed so far, our finding that participant-level parameter estimates from hierarchical Bayesian models result in biased statistical tests seems to be squarely at odds with other authors’ findings that such Bayesian estimates are better able to recover participants’ true parameter values than non-hierarchical methods. For example, Farrell and Ludwig (2008) found in their simulation study that hierarchical Bayesian methods provided estimates of participants’ ex-Gaussian parameters that were closest to the data-generating parameter values (see also Rouder et al., 2003). There are two likely reasons for these divergent results. Firstly, whereas Farrell and Ludwig were concerned with parameter estimation, we are concerned with statistical testing. In parameter estimation, the quantity of interest is the absolute deviation between the estimated and the true parameter values, which might very well be minimal for hierarchical Bayesian estimates. In statistical testing, on the other hand, it is not only the absolute deviation but also its direction that is of interest. If the estimated parameters systematically deviate from the true values in the direction of the group mean, estimates of the group-level variance that are based on such parameter estimates will systematically be too small, and will thus bias test statistics.

A second reason for the discrepancy with Farrell and Ludwig (2008) might lie in the relatively low degree of shrinkage in their study. The most extreme case simulated in Farrell & Ludwig’s study was an experiment with 80 participants and 20 trials per participant, whereas the most extreme case in our study was an experiment with 60 participants and two trials per participant. Consequently, the sampling variance was much greater in our study so that the participant-level estimates were strongly shrunken. Although it might be argued that such extreme cases are rarely encountered in practice, it should be noted that the model with normal distributions on all hierarchical levels considered here is extraordinarily well behaved and can usually be fitted reasonably well with only little data. More complex models, especially ones that rely heavily on the precise estimation of variance parameters (e.g., Ratcliff & Russ, in press), might show a problematic sensitivity to shrinkage for much larger sample sizes, a problem that should be explored in future studies.

To sum up, our simulation study showed that taking shortcut strategies for applying cognitive models to hierarchical data biases frequentist as well as Bayesian statistical tests; these biases are most pronounced when only little data is available. We therefore recommend that researchers avoid taking shortcuts and use hierarchical models to analyze hierarchical data.

## Footnotes

1 For convenience we assume that the participant-level variance is constant across participants. This assumption will be relaxed for our simulations reported below.

2 More specifically, because the effect size *δ* depends on the means of the two experimental groups, *μ*_1_,*μ*_2_ and the group-level variance *τ*^2^, the set Θ contains only one of the two means and the group-level variance.

3 Note that the nesting discussed here only refers to the participant means and group means. Researchers might also consider nesting participant- and group-specific variances. However, such nested variances might cause convergence problems in complex models if insufficient data are available.

4 This truncation was necessary because when fitting log-RTs, negative values of the group-level mean would imply impossibly small RTs. Nevertheless, due to the large mean of the prior and the comparatively small standard deviation, the effect of the truncation on our model fits was negligible.
